# Parenting Stress and Emotional/Behavioral Problems in Adolescents with Primary Headache

**DOI:** 10.3389/fneur.2017.00749

**Published:** 2018-01-19

**Authors:** Francesca Felicia Operto, Francesco Craig, Antonia Peschechera, Roberta Mazza, Paola Alessandra Lecce, Lucia Margari

**Affiliations:** ^1^Basic Medical Sciences, Neuroscience and Sense Organs Department, University of the Study of Bari “Aldo Moro”, Azienda Ospedaliero-Universitaria Consorziale Policlinico di Bari, Bari, Italy; ^2^Scientific Institute I.R.C.C.S. “Eugenio Medea” – “La Nostra Famiglia”, Brindisi, Italy

**Keywords:** primary headache, adolescents, parenting stress, behavioral problems, emotional problems

## Abstract

Primary headache is a frequent and disabling disorder, common among children and adolescents, and it is a painful syndrome often accompanied by functional impairment and associated with emotional and behavior problems. The aim of this study was to investigate parenting stress and emotional/behavioral problems in adolescents affected by primary headache compared with healthy adolescents. The study population consisted of 35 adolescents and a control group of 23 healthy subjects. The assessment included the administration of clinical standardized scales such as Parent Stress Index-Short Form, Pediatric Migraine Disability Assessment Score Questionnaire, and Child Behavior Checklist (CBCL). Headache group and control group did not differ in terms of parenting stress (*p* = 0.29). On the contrary, headache group showed more internalizing problems (*p* = 0.023), affective problems (*p* = 0.01), anxious (*p* = 0.001), and somatic complaints (*p* < 0.001) compared with control group. In addition, we found a significant correlation between PSI domains and specific CBCL subscales in the headache group. The findings emphasize the need for expanded intervention in the clinical treatment of pediatric headache, a treatment that may also include the family members. Further research is needed.

## Introduction

Headache is the most frequent neurological symptom and commonest manifestation of pain in childhood, representing one of the most frequent reasons for child neuropsychiatric consultation (1). A recent review estimated overall average prevalence of headache among children and adolescents was 54.4% (range: 43.1–65.8), and it resulted more common in female patients (2).

Pediatric headache, with its recurrent crises and the need for recurrent medical examinations could have a negative impact on family well-being (3). In literature, there are only two reports concerning the impact of primary headache on parenting stress: Esposito et al. (4) reported that parents of children with headache have higher stress levels on all of the three dimensions of PSI compared with parents of a control group, whereas Barone et al. showed no difference between headache group and control group for parenting stress (3). Moreover, parents of children affected by physical and psychiatric chronic illness seem to have higher stress levels. Esposito et al. (5) found higher scores of PSI in mothers of children affected by neurofibromatosis type 1, highlighting the importance of considering the environmental aspects of a chronic disease management in developmental age. More recently, Gatta et al. (6) showed higher stress levels in parents of children affected by psychiatric disease compared with parents of a control group and described an increase of the Parenting Stress Index associated with the severity of the disease.

In the past few years, many studies focused their attention on the presence of emotional/behavioral problems in children affected by primary headache, but limited data are available on the relationship between these problems and parenting stress. A previous meta-analysis had already described greater increase in internalizing than in externalizing problems in children and adolescents with chronic physical illness, including headache (7).

The role of emotional and behavioral problems in determining the perception of parenting stress should be better investigated; in turn, parenting stress becomes a part of a vicious cycle effect (8), thus increasing the risk for subsequent emotional and behavioral problems in children (9). Investigating the influence of children’s problems on the primary caregiver’s stress might supply a starting point for developing strategies to support this pediatric population and its families.

Investigating parenting stress and emotional/behavioral problems in adolescents affected by primary headache compared with healthy adolescents was the aim of this study. Another aim was to study the impact of headache severity on parenting stress and emotional/behavioral problems in adolescents with headache.

## Materials and Methods

### Subjects

In the past 6 months, we consecutively recruited 35 adolescents admitted to the Child and Adolescence Neuropsychiatry Unit—“Aldo Moro” University of Bari—affected by primary headache. The inclusion criteria for the study were (a) age between 11 and 18 years; (b) diagnosis of primary headache (with/without aura and tension-type) according to the International Classification of Headache Disorders (ICHD-3 beta version) (10). The exclusion criteria were the presence of secondary headache attributed to trauma or injury, vascular disorder, infection, or psychiatric disorder. The control group included 23 healthy adolescents, randomly recruited among students attending Secondary and Higher school of the same urban area. The small sample size may be justified by the general difficulty in recruiting healthy subjects.

For this study was not required an ethical review process by the Local Ethics Committee of Azienda Ospedaliero-Universitaria Policlinico di Bari (Italy) since all the procedures within the study assessment are included in the headache diagnostic protocol of our Child and Adolescence Neuropsychiatry Unit. All the participants were recruited after obtaining a written informed consent by their parents.

### Assessment

Headache assessment included the administration of the Pediatric Migraine Disability Assessment Score Questionnaire (PedMIDAS) and the administration of a questionnaire created *ad hoc* in order to assess the following characteristics: disease duration (expressed in years) and frequency of the attacks (number of events/month). In both headache group and control group, we administered the Italian version of Parenting Stress Index-Short Form, in order to investigate the perceived stress in the parents of children with primary headache and the Italian version of the Child Behavior Checklist questionnaire (CBCL) to assess the psychological and social competence of children and their psychological and behavioral problems.

The PedMIDAS (11) is the only specific measure of headache-related disability in the pediatric population. The measure quantifies headache-related disability during the past 3 months. Responses are summed and then graded as reflecting little to none (0–10, Grade I), mild (11–30, Grade II), moderate (31–50, Grade III), and severe disability (further than 50, Grade IV).

The Parent Stress Index-Short Form (PSI-SF) (12) is a standardized tool, which yields scores of parenting stress across four domains: parenting distress (PD), difficult child (DC), parent-child dysfunctional interaction (P-CDI), and total stress (TS). It has 36 items and provides both raw and percentile scores. Each item was graded on a 5-point Likert scale. The PD scale defines the level of distress which a parent perceives in his role, linked to personal factors. The DC scale values how much the parent perceives his child as easy/difficult to manage, considering some of his behavioral characteristics. The P-CDI scale values the parental perception of a child who does not respond to the family expectations and of an interaction neither reinforcing nor rewarding with the child. The TS scores, obtained by the sum of the scores of the three subscales, can be interpreted as a stress index related to the only parenting role. The test includes also a defensive responding scale, useful to control the validity of the protocol, which indicates if the parent tends to give a better self-image, minimizing the problems and the perceived stress in the relationship with the child. Higher scores indicate higher perceived stress in the parents. The subscale scores range from 12 to 60, and the TS score ranges from 36 to 180, with higher scores indicating greater levels of parenting stress. Thus, responses higher than the 85th percentile (1 SD above the average) are interpreted as “clinically significant” for high levels of family stress.

The CBCL (13) is a rating scale available for assessing behavioral or emotional problems and social competence in children aged 6–18 years. It is a parent completed survey: parents/caregivers are instructed to answer questions about their child’s behavior during the past 6 months. Items are scored as follows: 0 = not true (as far as you know), 1 = somewhat or sometimes true, or 2 = very true or often true. In this study, we focused our attention on some of internalizing (affective problems, anxious problems, and somatic complaints) and externalizing problems [attention-deficit/hyperactivity disorder (ADHD), Oppositional defiant disorder (ODD), conduct disorder (CD)]. For CBCL, the borderline (*t*-score >65) and clinical (*t*-score >70) scores were put together. For each group, a clinical cutpoint for the domain-specific syndrome scales and broadband scales is determined as the minimum raw score corresponding with *T*-score ≥65.

### Data Analysis

All demographic and clinical variables went through statistical analysis. Descriptive analysis was conducted for socio-demographics featuring two samples. In order to compare age and gender between the headache and control groups, we used respectively Mann–Whitney *U* test and Fisher’s exact test. The Mann–Whitney *U* test is a non-parametric test, and it was used to examine the difference of PSI-SF and CBCL scores between headache and control groups. In addition, a correlation analysis was also conducted for both groups to evaluate the relationship between parenting stress (PSI-SF) and internalizing/externalizing problems (CBCL). Spearman’s rho (*r*) was used to describe the strength and direction of relationship between variables. A *p*-value of less than 0.05 was considered as statistically significant. For statistical processing, we used the Statistical Package for Social Science version 20.0.

## Results

The mean age of headache group was 14.89 years (SD = 3.2), while the mean age of control group was 14.75 years (SD = 3.17). In the headache group, 15 patients were male (42%) and 20 were female (58%), while in the control group 9 adolescents were male (39%) and 14 were female (61%). The results of the demographic data showed no significant difference between the two groups in gender (*p* = 0.32) and age (*p* = 0.26).

An evaluation of headache severity using PedMIDAS (Grades I–IV) detected that in the headache group 28.6% of adolescents showed low disability, 20% showed mild disability, 31.4% showed moderate disability, and 20% showed severe disability.

### Differences in PSI-SF and CBCL Scores in the Headache and Control Groups

Means and SDs for control and headache groups are reported in Table 1. The two groups did not differ in terms of parenting stress.

**Table 1:** Significant differences in PSI scores between groups.

Headache (*n* = 35)			Control (*n* = 23)			
	% >Cut-off	M ± SD	% >Cut-off	M ± SD	*F*	*p*-Value
PD	14	24.2 ± 8.3	3	22.2 ± 6.8	332.5	0.26
DC	28	27.3 ± 9.4	5	25.3 ± 11.3	39.5	0.84
P-CDI	26	21.8 ± 1.3	4	2.5 ± 9.6	396.7	0.92
DR	–	15.5 ± 6.2	–	12.8 ± 4.3	302.2	0.1
TS	22	73.2 ± 24.5	3	67.3 ± 22.3	337.2	0.29

*PD, parenting distress; DC, difficult child; P-CDI, parent-child dysfunctional interaction DR, defensive responding; TS, total stress*.

We analyzed the difference in CBCL scores between the two groups, as shown in Table 2. The finding reveals a statistically significant difference between adolescents affected by headaches and the control group in internalizing problems (*p* = 0.023), a statistically significant difference in affective problems (*p* = 0.01), anxious (*p* = 0.001), and somatic complaints (*p* < 0.001) between headache group and control group.

**Table 2:** Significant differences in CBCL scores between groups.

Headache (*n* = 35)			Control (*n* = 23)			
	% >Cut-off	M ± SD	% >Cut-off	M ± SD	*F*	*p*-Value
Internalizing	48	6.8 ± 11.2	13	53.2 ± 14.5	260.0	0.023*
Externalizing	14	5.7 ± 11.9	30	5.2 ± 1.4	374.5	0.65
Total problems	25	56.9 ± 11.9	17	52.6 ± 9.5	316.2	0.17
Affective problems	31	57.2 ± 9.5	26	45.2 ± 17.5	258.5	0.01*
Anxious	34	61.2 ± 8.7	31	46.8 ± 12.3	187.0	0.001*
Somatic complaints	42	63.9 ± 9.3	13	39.6 ± 14.6	72.4	<0.001*
ADHD	10	54.5 ± 7.1	8	53.6 ± 6.3	385.5	0.78
ODD	8	54.11 ± 8.3	6	5.6 ± 13.4	309.5	0.13
CD	8	52.4 ± 11.5	8	51.4 ± 11.6	360.0	0.47

*CBCL, child behavior checklist; ADHD, attention-deficit/hyperactivity disorder; ODD, oppositional defiant disorder; CD, conduct disorder*.

*p < 0.05.

### Correlation between Headache Disability, Parenting Stress, and Emotional/Behavioral Problems Scores in the Headache and Control Groups

Table S3 in Supplementary Material shows bivariate associations between frequency of the attacks, disease duration, PedMIDAS, PSI-SF, and CBCL scores in the headache group.

Frequency of the attacks was positively correlated with internalizing problems (*p* = 0.024), total problems (*p* = 0.019), PD (*p* = 0.01), DC (*p* = 0.036), P-CDI (*p* = 0.002), and TS (*p* = 0.003) scores. A high positive correlation was found between PedMIDAS scores and somatic complaints (*p* = 0.004). We found a positive correlation between DC scores and internalizing problems (*p* = 0.002), externalizing problems (*p* = 0.007), total problems (*p* = 0.001), affective problems (*p* = 0.003), somatic complaints (*p* = 0.04), ADHD (*p* = 0.02), ODD (*p* = 0.014), and CD (*p* = 0.007). We found a positive correlation between P-CDI scores and internalizing problems (*p* = 0.004), externalizing problems (*p* = 0.019), total problems (*p* = 0.03), affective problems (*p* = 0.004), ADHD (*p* = 0.003), ODD (*p* = 0.003), and CD (*p* = 0.002). We found a positive correlation between TS scores and internalizing problems (*p* = 0.001), externalizing problems (*p* = 0.004), total problems (*p* = 0.004), affective problems (*p* = 0.004), ADHD (*p* = 0.03), ODD (*p* = 0.019), and CD (*p* = 0.02).

As showed in Table S4 in Supplementary Material, no statistical correlation between PSI-SF and CBCL scores were found in the control group.

## Discussion

Headache is a disabling condition not only for adolescents but also for their parents; with its recurrent crises and the need for recurrent medical examinations, headache can have a negative impact on family well-being (3). In this study, we evaluated parenting stress and emotional/behavioral problems in adolescents affected by primary headache, compared with a group of healthy adolescents.

Our results have not detected statistically significant difference in terms of parenting stress between our two groups. In literature, there are two reports concerning the impact of primary headache on parenting stress, using PSI as psychodiagnostic tool. They provide conflicting evidences. In 2013, Esposito et al. reported that parents of 218 children with migraine without aura had higher stress levels on all of the three dimensions of PSI compared with parents of a control group (4). Whereas, in 2015, Barone et al. compared 71 school-aged children with headache and 71 children from a low-risk normative population, showing no difference between headache group and control group for maternal stress. Furthermore, children’s behavioral problems were associated with higher maternal stress (3).

In the context of emotional and behavioral problems, a meta-analysis which used CBCL as psychodiagnostic tool in order to explore the presence of internalizing and externalizing symptoms found that more psychopathological symptoms were present in patients with primary headache, and the difference was more marked in the area of internalizing disorders (14). Previous studies detected that children with migraine were significantly more likely to have abnormalities in somatic, anxiety–depressive, social and attention CBCL domains, compared with controls (15–17). Some authors hypothesized that life events and their psychological processing are fundamental in the etiopathogenesis of primary headache in children and adolescents and that they can act in two different ways: either as predisposing a chronic state of anxiety or depression (even subclinical) or as triggering a cascade of psychological events which, in turn, activate the biological mechanisms of migraine attack (18). Margari et al. found, first, that patients with migraine and tension-type headache had more psychopathological symptoms than healthy controls as well as, second, that the difference was higher with regard to internalizing problems. They suggested that the relationship between migraine and psychopathology may be due to the involvement of the same neurotransmitters (19). According to previous literature data, we found a statistically significant difference in affective problems, anxious and somatic complaints between headache group and control group. These results confirmed that there is a large emotional component linked to headache, and that headache is a complex biomedical disorder in which psychological factors may play a contributory maintaining and/or reactive role.

Another aim of this study was to investigate the correlation between PedMIDAS, PSI-SF, and CBCL scores in the headache and control groups. We found a statistical significant correlation between PedMIDAS scores and somatic complaints and between PSI domains and specific CBCL subscales in the headache group. Frequency of headache attacks was associated with increasing parenting stress, indicating that headache frequency, rather than disease duration, increases the risk of parenting stress. This result seems to be consistent with other researches which found that parents of children with chronic disease have high levels of parenting stress (5, 20). This result suggests the importance of informing parents about the characteristics of headache, e.g., the frequency of attacks and associated symptoms. Using familiar coping strategies could help parents reduce their stress levels before and/or during migraine attacks.

In addition, as already reported by Barone et al. (3), we found that high internalizing and externalizing scores correspond to worsened parenting stress across DC and parent–child dysfunctional interaction domains. These findings suggest that emotional and behavioral problems in adolescents with headache are significant sources of parenting stress. What cannot be ascertained, however, due to the correlational nature of this investigation, is whether these same-child-and-parent factors cause parenting stress. Despite the uncertainties about the cause and direction of internalizing and externalizing symptoms in primary headache disorders, the treatment by the clinicians confronted with young headache patients should take into account that comorbidity among children and adolescents with headache poses an extra load on parenting stress and family quality of life.

Certain methodological limitations in this study should be noted. The small sample size may be justified by the general difficulty in recruiting healthy subjects; the relatively small study sample did not allow the generalization of findings and did not allow analyses for all the subtypes of primary headache.

In conclusion, we did not find a difference in parenting stress between headache group and control group; nevertheless, we found that high internalizing and externalizing scores in adolescents with headache correspond to worsened parenting stress across domains of DC and parent–child dysfunctional interaction. The findings emphasize the need for an intervention that includes all the family members in the clinical treatment of pediatric headache. Further research is needed.

## Ethics Statement

All the participants were recruited after obtaining a written informed consent by their parents. For this study was not required an ethical review process by the Local Ethics Committee of Azienda Ospedaliero-Universitaria Policlinico di Bari (Italy) since all the procedures within the study assessment are included in the headache diagnostic protocol of our Child and Adolescence Neuropsychiatry Unit.

## Author Contributions

LM, FFO, and PAL contributed to the conception, the design, the supervision, and the final approval of the work. AP and RM contributed to the enrollment of the participants and performed the general and cognitive assessment and the data collection. FC performed statistical analyses.

## Conflict of Interest Statement

The authors declare that the research was conducted in the absence of any commercial or financial relationships that could be construed as a potential conflict of interest.

## References

[1] Abu-Arafeh I Razak S Sivaraman B Graham C. Prevalence of headache and migraine in children and adolescents: a systematic review of population-based studies. Dev Med Child Neurol (2010) 52(12):1088–97. 10.1111/j.1469-8749.2010.03793.x 20875042

[2] Wober-Bingol C. Epidemiology of migraine and headache in children and adolescents. Curr Pain Headache Rep (2013) 17(6):341. 10.1007/s11916-013-0341-z 23700075

[3] Barone L Lionetti F Dellagiulia A Galli F Molteni S Balottin U Behavioural problems in children with headache and maternal stress: is children’s attachment security a protective factor? Inf Child Dev (2015) 25:502–15. 10.1002/icd.1950

[4] Esposito M Gallai B Parisi L Roccella M Marotta R Lavano SM Maternal stress and childhood migraine: a new perspective on management. Neuropsychiatr Dis Treat (2013) 9:351–5. 10.2147/NDT.S42818 23493447 PMC3593768

[5] Esposito M Marotta R Roccella M Gallai B Parisi L Lavano SM Pediatric neurofibromatosis I and parental stress: a multi center study. Neuropsychiatr Dis Treat (2014) 10:141–6. 10.2147/NDT.S55518 24489471 PMC3904813

[6] Gatta M Balottin L Mannarini S Birocchi V Del Col L Battistella PA Parental stress and psychopathological traits in children and adolescents. A controlled study. Riv Psichiatr (2016) 51(6):251–9. 10.1708/2596.26726 27996985

[7] Pinquart M Shen Y. Behavior problems in children and adolescents with chronic physical illness: a meta-analysis. J Pediatr Psychol (2011) 36:1003–16. 10.1093/jpepsy/jsr042 21810623

[8] Neece CL Green SA Baker BL Parenting stress and child behaviour problems: a transactional relationship across time. Am J Intellect Dev Disabil (2012) 117:48–66. 10.1352/1944-7558-117.1.48 22264112 PMC4861150

[9] Tharner A Luijk MP van Ijzendoorn MH Bakermans-Kranenburg MJ Jadooe VW Hofman A Infant attachment, parenting stress, and child emotional and behavioral problems at age 3 years. Parent Sci Pract (2012) 12:261–81. 10.1080/15295192.2012.709150

[10] Headache Classification Committee of the International Headache Society (IHS). The International Classification of Headache Disorders, 3rd edition (Beta Version). Cephalalgia (2013) 33(9):629–808. 10.1177/0333102413485658 23771276

[11] Hershey AD Powers SW Vockell AL LeCates SI Segers A Kabbouche MA. Development of a patient-based grading scale for PedMIDAS. Cephalalgia (2004) 15:844–89. 10.1111/j.1468-2982.2004.00757.x 15377315

[12] Abidin RR Parenting Stress Index, Fourth Edition (“PSI-4”). Lutz, FL: Psychological Assessment Resources (2012).

[13] Achenbach TM Rescorla LA Manual for the ASEBA School-Age Forms and Profiles. Burlington, VT: University of Vermont, Research Center for Children, Youth, and Families (2001).

[14] Balottin U Fusar Poli P Termine C Molteni S Galli F. Psychopathological symptoms in child and adolescent migraine and tension-type headache: a meta-analysis. Cephalalgia (2013) 33(2):112–22. 10.1177/0333102412468386 23203505

[15] Galli F D’Antuono G Tarantino S Viviano F Borrelli O Chirumbolo A Headache and recurrent abdominal pain: a controlled study by the means of the child behaviour checklist (CBCL). Cephalalgia (2007) 27(3):211–9. 10.1111/j.1468-2982.2006.01271.x 17381555

[16] Vannatta K Getzoff EA Powers SW Noll RB Gerhardt CA Hershey AD. Multiple perspectives on the psychological functioning of children with and without migraine. Headache (2008) 48(7):994–1004. 10.1111/j.1526-4610.2007.01051.x 18705025

[17] Arruda MA Bigal ME. Migraine and migraine subtypes in preadolescent children: association with school performance. Neurology (2012) 79:1881–8. 10.1212/WNL.0b013e318271f812 23109652

[18] Balottin U Chiappedi M Rossi M Termine C Nappi G. Childhood and adolescent migraine: a neuropsychiatric disorder? Med Hypotheses (2011) 76(6):778–81. 10.1016/j.mehy.2011.02.016 21356578

[19] Margari F Lucarelli E Craig F Petruzzelli MG Lecce PA Margari L. Psychopathology in children and adolescents with primary headaches: categorical and dimensional approaches. Cephalalgia (2013) 33(16):1311–8. 10.1177/0333102413495966 23827982

[20] Cousino MA Hazen RA. Parenting stress among caregivers of children with chronic illness: a systematic review. J Pediatr Psychol (2013) 38(8):809–28. 10.1093/jpepsy/jst049 23843630

