# Supplementary material for: Parenting Stress and Emotional/Behavioral Problems in Adolescents with Primary Headache
Source: Front Neurol. 2018 Jan 19;8:749. doi: 10.3389/fneur.2017.00749 (PMC5780397; doi:10.3389/fneur.2017.00749)
Supplement: Supplementary file 1 [file table_3.doc]

Supplementary Material

**Parenting Stress and Emotional/ Behavioral Problems in Adolescents with Primary Headache**

Francesca Felicia Operto, Francesco Craig, Antonia Peschechera, Roberta Mazza, Paola Alessandra Lecce, Lucia Margari*.

*** Correspondence:**

Prof. Lucia Margari

lucia.margari@uniba.it

Supplementary data: table 3

| **Table 3. Bivariate correlations between parenting stress, children’s internalizing and externalizing problems in the headache group (n = 35)** | | | | | | | | | | | | | |
| --- | --- | --- | --- | --- | --- | --- | --- | --- | --- | --- | --- | --- | --- |
|  | | **Frequency** | **Duration** | **PedMIDAS** | **Internalizing** | **Externalizing** | **Total Problems** | **Affective Problems** | **Anxious** | **Somatic Complain** | **ADHD** | **ODD** | **CD** |
| Frequency | r | - | .001 | .322 | .382 | .283 | .394 | .309 | .291 | .221 | .079 | .175 | .249 |
|  | p | - | .996 | .059 | .024* | .099 | .019* | .071 | .09 | .205 | .652 | .314 | .151 |
| Duration | r | .001 | - | .319 | .212 | .301 | .288 | .284 | .182 | .554 | .266 | .301 | .256 |
|  | p | .996 | - | .062 | .221 | .079 | .093 | .098 | .295 | .001** | .122 | .221 | .021 |
| PedMIDAS | r | .322 | .319 | - | .182 | .134 | .194 | .215 | .208 | .472 | .011 | .185 | .155 |
|  | p | .059 | .062 | - | .295 | .442 | .264 | .215 | .230 | .004** | .949 | .288 | .373 |
| PD | r | .431 | .061 | -.193 | .327 | .185 | .188 | .233 | .040 | .020 | .107 | .125 | .099 |
|  | p | .01** | .727 | .265 | .055 | .288 | .279 | .178 | .821 | .910 | .540 | .475 | .573 |
| DC | r | .356 | .265 | .188 | .497 | .545 | .570 | .492 | .211 | .329 | .372 | .411 | .449 |
|  | p | .036* | .123 | .281 | .002** | .007** | .001** | .003** | .224 | .046* | .02* | .014* | .007** |
| P-CDI | r | .506 | .225 | .085 | .473 | .393 | .360 | .558 | .116 | .278 | .480 | .481 | .489 |
|  | p | .002** | .193 | .625 | .004** | .019* | .03* | .004** | .507 | .105 | .003** | .003** | .002** |
| DR | r | .406 | .036 | -.179 | .407 | .137 | .233 | .137 | .103 | .087 | .271 | .278 | .297 |
|  | p | .062 | .838 | .303 | .062 | .434 | .178 | .434 | .555 | .619 | .116 | .106 | .084 |
| TS | r | .489 | .232 | .041 | .528 | .465 | .471 | .464 | .134 | .262 | .354 | .393 | .382 |
|  | p | .003** | .180 | .815 | .001** | .004** | .004** | .004** | .442 | .128 | .03* | .019* | .02* |
| Parenting Distress (PD), Difficult Child (DC), Dysfunctional Interaction Parent-Child (P-CDI), Defensive Responding (DR), Total Stress (TS);*p<.05. **p<.01 | | | | | | | | | | | | | |
