# Supplementary material for: Parenting Stress and Emotional/Behavioral Problems in Adolescents with Primary Headache
Source: Front Neurol. 2018 Jan 19;8:749. doi: 10.3389/fneur.2017.00749 (PMC5780397; doi:10.3389/fneur.2017.00749)
Supplement: Supplementary file 2 [file table_4.doc]

Supplementary Material

**Parenting Stress and Emotional/Behavioral Problems in Adolescents with Primary Headache**

Francesca Felicia Operto, Francesco Craig, Antonia Peschechera, Roberta Mazza, Paola Alessandra Lecce, Lucia Margari*.

*** Correspondence:**

Prof. Lucia Margari

lucia.margari@uniba.it

Supplementary data: table 4.

| **Table 4. Bivariate correlations between parenting stress, children’s internalizing and externalizing problems in the control group (n = 23)** | | | | | | | | | | |
| --- | --- | --- | --- | --- | --- | --- | --- | --- | --- | --- |
|  | | **Internalizing** | **Externalizing** | **Total Problems** | **Affective Problems** | **Anxious** | **Somatic Complain** | **ADHD** | **ODD** | **CD** |
| PD | r | .175 | -.356 | -.055 | .186 | .190 | .052 | .267 | -.030 | .297 |
|  | p | .423 | .095 | .805 | .395 | .384 | .814 | .219 | .892 | .168 |
| DC | r | .010 | -.010 | -.010 | -.018 | -.005 | -.107 | .055 | .207 | .053 |
|  | p | .964 | .964 | .965 | .934 | .982 | .626 | .804 | .342 | .810 |
| P-CDI | r | -.078 | .038 | .300 | -.157 | -.117 | -.183 | .167 | -.003 | .161 |
|  | p | .722 | .864 | .164 | .474 | .595 | .404 | .447 | .991 | .464 |
| DR | r | .145 | .078 | .047 | -.086 | -.062 | -.072 | .144 | .096 | .081 |
|  | p | .509 | .722 | .833 | .696 | .778 | .745 | .512 | .662 | .715 |
| TS | r | -.007 | -.342 | .050 | .002 | .012 | -.080 | .192 | -.020 | .238 |
|  | p | .976 | .111 | .823 | .995 | .958 | .715 | .380 | .926 | .275 |
| Parenting Distress (PD), Difficult Child (DC), Dysfunctional Interaction Parent-Child (P-CDI), Defensive Responding (DR), Total Stress (TS); | | | | | | | | | | |
